# Supplementary material for: Evolution of cooperation in an epithelium
Source: J R Soc Interface. 2019 Mar 27;16(152):20180918. doi: 10.1098/rsif.2018.0918 (PMC6451401; doi:10.1098/rsif.2018.0918)
Supplement: Varying the spring constant in the Voronoi Tessellation model [file rsif20180918supp1.pdf]

Evolution of cooperation on an epithelium
Supplementary Information

Jessie Renton* Karen M. Page
Department of Mathematics, University College London,
Gower Street, London WC1E 6BT, UK

Varying μ in the Voronoi Tessellation model

In the main text we used parameter values for the Voronoi Tessellation model taken from [1] as shown in Table 2. Here we show that our main result, that the decoupling of birth and death promotes cooperation, is robust to changes in these values. In particular we look at changes in the ratio μ/ν, which determines how quickly relaxation occurs when the system is out of equilibrium (i.e. a birth or death has occurred). Varying this ratio while keeping the birth and death rates, λ, constant, will alter the tissue dynamics and topology. This is clear from Figure 1, which shows the polygon (neighbour number) distributions for different values of μ, while keeping $\nu = 1$ constant. Decreasing μ leads to more variation in neighbour number.

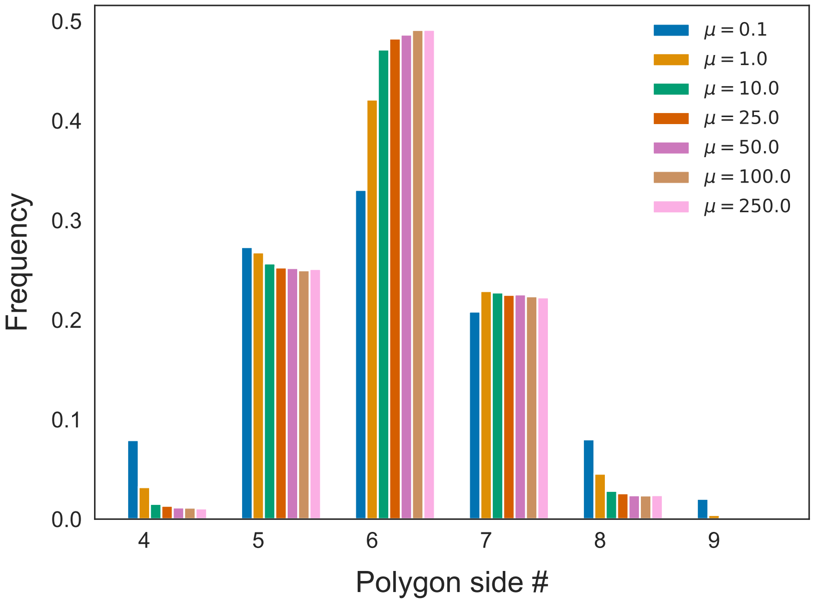

Figure 1: Polygon distributions for various values of μ. In the main text we use $\mu = 50$. Decreasing μ below this value leads to increased variation in side number around the mean. Each distribution is calculated from data collected from three simulation runs of 100 hours simulation time.

*Corresponding author: jessica.renton.16@ucl.ac.uk

To consider the effect of varying μ we calculate the fixation probability for a range of values using the approximate analytical technique described in Section 3.2 of the main text. We calculate Λ_n^{CC} (Equation (16)) computationally by running 500 simulations for each μ. The fixation probabilities are then calculated using Equation (18) and the critical benefit-to-cost ratios, $(b/c)^*$, using Equation (19). These are plotted in Figure 2 and Figure 3 respectively. We keep all other parameters constant with the exception of Δt which is set to be

$$\Delta t = 0.005 \times \min\left(1, \frac{50}{\mu}\right) . \tag{1}$$

This ensures that when μ is increased, Δt remains small enough for numerical stability.

Figure 3 shows that $(b/c)^*$ increases with μ. Increasing μ above 50 (the value used in the main text) leads to small increases in $(b/c)^*$, but it remains well below the values for death-birth updating (see Table 1). Decreasing μ below 50 leads to fast decreases in $(b/c)^*$, thus the success of cooperation is increased. It is not possible without further investigation to ascertain whether this is due to the changes in graph topology, or whether there are other effects to the tissue dynamics which are promoting cooperation. Note that we do not claim this range of μ to be biologically reasonable, indeed for low values of μ the forces will act so slowly that it certainly is not realistic. We have chosen to show this large range of μ in order to demonstrate the robustness of our result that decoupling birth and death promotes cooperation in the Voronoi Tessellation model.

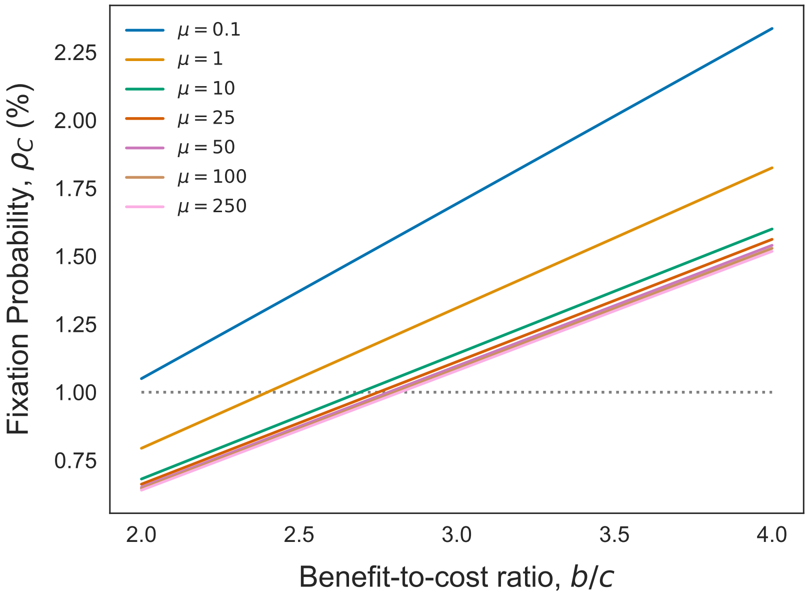

Figure 2: Fixation probabilities for a cooperative mutant in the Voronoi Tessellation model with decoupled birth and death plotted for different values of the spring constant μ. The dotted line shows the neutral fixation probability, $\rho_0 = 1/N$, where $N = 100$ is the population size. The point where $\rho_C = \rho_0$ is the critical benefit-to-cost ratio $(b/c)^*$ plotted in Figure 3. For $\rho_C > \rho_0$ cooperation is beneficial, thus cooperative success increases as the spring constant is decreased.

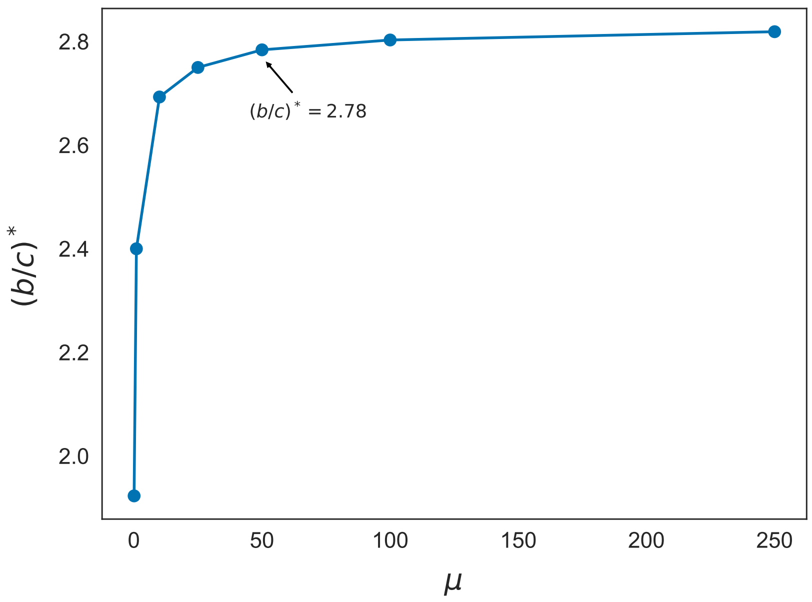

Figure 3: Critical benefit-to-cost ratio, $(b/c)^*$, against the spring constant, μ, for a cooperative mutant in the Voronoi Tessellation model with decoupled birth and death. The labelled point corresponds to the value of μ used in simulations in the main text. Lower values of $(b/c)^*$ imply that cooperation is more successful, thus cooperative success is decreasing with μ.

References

[1] J. M. Osborne, A. G. Fletcher, J. M. Pitt-Francis, P. K. Maini, and D. J. Gavaghan. Comparing individual-based approaches to modelling the self-organization of multicellular tissues. *PLOS Computational Biology*, 13(2):e1005387, 2017. doi: 10.1371/journal.pcbi.1005387.
